# An investigation of metabolome in blood in patients with chronic peripheral, posttraumatic/postsurgical neuropathic pain

**DOI:** 10.1038/s41598-022-26405-6

**Published:** 2022-12-15

**Authors:** Bijar Ghafouri, Katarina Thordeman, Romina Hadjikani, Anders Bay Nord, Björn Gerdle, Emmanuel Bäckryd

**Affiliations:** 1 Pain and Rehabilitation Center, and Department of Health, Medicine and Caring Sciences, Linköping University, 581 85 Linköping, Sweden; 2 Swedish NMR Centre at the University of Gothenburg, Gothenburg, Sweden

**Keywords:** Metabolomics, Diagnostic markers

## Abstract

Neuropathic pain (NP) is a chronic pain condition resulting from a lesion or disease in the somatosensory nervous system. The aim of this study was to investigate the metabolome in plasma from patients with chronic peripheral, posttraumatic/postsurgical NP compared to healthy controls. Further, we aimed to investigate the correlation between pain intensity and the metabolome in plasma. The metabolic profile in plasma samples from 16 patients with chronic NP and 12 healthy controls was analyzed using a nuclear magnetic resonance spectroscopy method. Information about pain intensity, pain duration, body mass index (BMI), age, sex, and blood pressure were obtained through a questionnaire and clinical examination. Multivariate data analysis was used to identify metabolites significant for group separation and their correlation with pain intensity and duration, BMI, and age. We found 50 out of 326 features in plasma significantly contributing to group discrimination between NP and controls. Several of the metabolites that significantly differed were involved in inflammatory processes, while others were important for central nervous system functioning and neural signaling. There was no correlation between pain intensity and levels of metabolite in NP. These findings indicate that there seems to be peripheral/systemic differences in the metabolic profile between patients with chronic NP and healthy individuals.

## Introduction

The prevalence of chronic pain with neuropathic characteristics has been approximated to be about 7–10% in the general population^1^; more frequent among women, people with lower professional status and patients > 50 years of age. Neuropathic pain (NP) is defined as pain caused by a lesion or disease of the somatosensory nervous system, by the International Association of the Study of Pain (IASP). NP is associated with typical symptoms such as a burning and electrical-like sensation, allodynia, and hyperalgesia^2^. These patients report various psychological distress such as depression, anxiety and sleep disorders to a greater extent than pain patients without neuropathic pain, also they experience a lower quality of life^3^. Peripheral and central sensitization are important pathophysiological mechanisms found in chronic NP but there is still lack of molecular knowledge behind the mechanisms and treatment is challenging^4,5^.

There is growing evidence that an interplay between the immune system and nervous system, i.e., neuroinflammation, plays a key role in NP^6,7^. Neuroinflammation because of neuronal injuries includes the activation of local immune cells and glial cells, alterations in capillary permeability and infiltration of peripheral leukocytes, leading to overproduction of different pro-inflammatory mediators that can modulate pain sensitivity and produce a long-lasting peripheral and central sensitization^8,9^.

Diagnosis is based on a process that includes a pain anamnesis, clinical examination covering a complete neurological examination and performance of diagnostic tests and reporting of pain. Neuropathic pain can be graded as possible, probable, or definite^5^. The treatment for NP is insufficient and only partially of patients are responsive to nearly all treatment^10^. Therefore, it is of great importance to get a deeper understanding of the molecular mechanisms underlying chronic NP to improve the diagnostic tools and to develop more target-specific drugs. Several potential mediators have found to be altered in chronic NP, spanning from altered expression of long non-coding RNA and inflammation activity to ion channel expression and activity^6,11–14^.

Metabolites are small organic molecules with a low molecular weight that are formed and transformed during metabolism. They represent the biochemical activity in the current environment. Metabolomics is an example of the field of biomarkers that may be a useful tool in research of chronic pain conditions^15,16^. Knowledge about different metabolic pathways that are activated during inflammation and their respective metabolites and how they react after treatments can be helpful e.g., for the development of new treatment strategies of hypersensitivity. Nuclear magnetic resonance (NMR) spectroscopy is a technique enabling metabolomic study^17–19^. With this spectroscopy method the detection of up to hundreds of metabolites is possible in complex biofluids such as urine or plasma.

Hence, the aim of this study was to analyze the metabolome in plasma and compare the metabolic profiles between chronic NP patients and healthy controls. Further, we aimed to investigate the correlation between pain intensity and the metabolome in plasma.

## Material and methods

### Patients

A cohort of 16 patients (males and females) with chronic NP were recruited to this study. All patients included in this study were recruited from a clinical trial where intrathecal bolus injections of ziconotide were administered to ascertain its analgesic effect, at the same time blood and CSF samples were collected^20^. Patients suffering from chronic peripheral, posttraumatic/postsurgical NP refractory to pharmacological treatment and under consideration for spinal cord stimulation (SCS), at Linköping University Hospital, Sweden, were invited to participate in the study. A medical examination was performed to get essential clinical data from the patients. Inclusion criteria for participation were: (1) patient must be at least 18 years of age, (2) patient suffering from chronic peripheral neuropathic pain (6 months or more) caused by trauma or surgery and had failed conventional pharmacological treatment, (3) average visual analogue pain scale intensity last week 40 mm or more, (4) patient capable of judgement, meaning the patient is able to comprehend information about the drug, its administration, and evaluation of efficacy and side effects, and (5) signed informed consent.

Exclusion criteria were: (1) Intrathecal chemotherapy, (2) pregnant or lactating women, (3) limited life expectancy, (4) intracranial hypertension, (5) known liver or kidney disease characterized by serum transaminases, total bilirubin, alkaline phosphatase or creatinine > 1.2 times above the upper normal limit, (6) advanced cardiopulmonary disease, (7) ongoing infection in the lumbar area (systematically or locally), (8) coagulopathy, (9) history of psychiatric disorder, (10) allergy to ziconotide or any of the excipients in the ziconotide vial, and/or (11) participation in another clinical trial during the last 30 days. Among the patients with chronic neuropathic pain the pain location differed such that 4 of these suffered from peripheral neuropathic pain in the upper extremity and 12 from peripheral neuropathic pain in the lower extremity. All patients included in this study described their pain as a continuous. Twelve of the patients experienced an exacerbation of the pain during physical activity. Moreover, 9 of the patients had other concomitant diseases besides their pain diagnosis; hypertension was found amongst 4 of these, polymyalgia rheumatica in 1 patient, psoriasis amongst (n = 2), fibromyalgia (n = 1), diabetes (n = 1), mild angina (n = 2), panic disorder (n = 1), mild obstructive lung disease (n = 1), vertebral compressions (n = 1), orthostatism (n = 1), peptic ulcer (n = 1), dyspepsia (n = 1) and anemia (n = 1). Nine patients also had a past medical history; where 2 patients had a history of depression, 1 had a history of alcohol abuse, 5 patients had undergone surgery at some point in life and 2 patients had been through a myocardial infarction earlier in life. All patients but one had either unsuccessfully tried tricyclics/duloxetine or had concomitant such medicines. All patients but one had also either unsuccefully tried gabapentinoids or had concomitant gabapentinoids. Moreover, all patients had either previously used or were using opioids. None had tried capsaicine and one had tried topical lidocaine (no patient had dynamic mechanical allodynia)^21^. The use of concomitant medication including analgesics was also reported: paracetamol, NSAID, weak opioids, strong opioids, gabapentin, antidepressants, capsaicin, and topical medication. Overall, 4 of the patients did not use any medication at all at inclusion, while 3 patients had a single medication and 9 patients used 2 drugs or more. The distribution of the usage of the different medications were paracetamol (n = 7), NSAID (n = 2), opioids (n = 9), gabapentin (n = 7) and antidepressants (n = 6). Furthermore, the International Classification of Diseases version 10 (ICD10) diagnosis for each patient was also registered, see Table 1.

**Table 1:** Overview of main cause of pain based on ICD10 classification system (n = 16).

ICD10 diagnosis	Description	Number of patients
S342	Injury of nerve root of lumbar or sacral spine	8
S740	Injury of sciatic nerve at hip and thigh level	1
S549	Injury of unspecified nerve at forearm level	1
S949	Injury of unspecified nerve at ankle or foot level	1
S841	Injury of peroneal nerve at lower leg level	1
S142	Injury of nerve root of cervical spine	3
S343	Injury of cauda equina	1
G629	Polyneuropathy, unspecified	1

### Healthy controls

Twelve healthy controls were recruited through local advertisement at Linköping University. Absence of ongoing chronic pain was fundamental to be included in the healthy control group. Six of the healthy controls used some form of concomitant medication. Two of these used low dose budesonides regularly. One control used a beta2-stimulating drug, when necessary, occasionally. One control used depot medroxyprogesterone acetate and another oral vitamin B12. Mild heart or vascular disease occurred in 1 healthy control, to which no medication was needed. In this group there was no use of pain-relieving medication. Informed consent was obtained before assessment of their medical condition.

### Ethics

Verbal and written information about the study was given to all the participants and written informed consent was thereafter obtained from all the participants. The study was conducted in accordance with the Helsinki Declaration and Good Clinical Practice. The Ethical Review Board Regional Ethics Committee in Linköping approved the study (Dnr M136-06 and Dnr 2012/94–32).

### Background data

All the participants answered questionnaires about pain and their state of health. These questionnaires included factors such as age, pain duration (measured in months), average pain intensity estimated through Pain Visual Analogue Scale (VAS), location of pain, characterization of pain (constant, intermittent or transitory), impact of physical activity on pain, concomitant diseases, and ongoing medication. In addition, patient data about length, weight and blood pressure was gathered through a clinical examination. Also, an ICD10 diagnosis code was set for every patient.

### Sample collection

Venous blood samples were collected in EDTA tubes before the injection of ziconotide. Samples were centrifuged, and the fraction of plasma divided into tubes containing 0.5 ml each. The plasma was then stored in − 86 °C.

### Metabolomics analysis

The plasma samples were prepared by mixing the plasma 1:1 with phosphate buffer (75 mM Na_2_HPO_4_, 4% NaN_3_, 2.32 mM TSP-d4, 10% D_2_O, pH 7.4) to a total volume of 220 µl. The mixture was transferred to 3 mm NMR tubes using a SamplePro L liquid handling robot (Bruker BioSpin, Rheinstetten, Germany).

The samples were analyzed at Swedish Nuclear Magnetic Resonance (NMR) Centre in Gothenburg using an Oxford 800 MHz magnet (Bruker BioSpin, Germany) equipped with a Bruker Avance III HD console, 3 mm TCI cryoprobe and a cooled Sample Jet auto sampler. 1D CPMG-edited experiment (pulse sequence 'zgespe') as well as 2D J-resolved spectra (pulse sequence 'jresgpprqf') were acquired for each sample. Experimental details available upon request. Data acquisition and processing of raw data, including Fourier transformation, phasing, baseline correction and referencing to TSPd4 of the 1D CPMG data was performed in TopSpin 3.5pl7 (Bruker BioSpin). The data obtained from NMR were annotated with the use of Chenomx 8.4 (Chenomx Inc, Edmonton, Canada), the 2D J-resolved data and publicly available spectral databases such as HMDB^22^. A criterion that a metabolite was present at detectable levels in at least 40% of the subjects in one group to be included in the statistical analysis was set.

### Univariate statistics

IBM SPSS Statistics (version 27.0, IBM, United States) was used for univariate analysis to detect eventual differences in several patient factors between the patient group and the control group, namely, age, BMI, VAS, pain duration, systolic blood pressure and diastolic blood pressure. Primarily, the different variables were tested with Shapiro Wilk’s test of normality. Depending on whether the variable was normally distributed, or not different tests were performed. For the variables that were normally distributed the analysis was made with a t-test and for the variables that were not normally distributed Mann Whitney *U* test was used. P-value ≤ 0.05 was considered significant.

### Multivariate statistics

The metabolomics data was statistically analyzed with SIMCA-P + (v.17.0, Sartorius Stedim Biotech, Umeå, Sweden) to study the correlation between concentration in plasma and group membership using Orthogonal Partial least squares discriminant analysis (OPLS-DA). Also, regression analyses of the identified metabolites to pain severity (VAS), age, BMI and pain duration were performed in the NP group using orthogonal partial least square analysis (OPLS). Before the OPLSA-DA and OPLS analysis, principal component analysis (PCA) was used to check for multivariate outliers. The parameters used for evaluation of the OPLS-DA and OPLS models were R^2^ (describes the goodness of fit) and Q^2^ (describes the goodness of prediction) with a difference between R^2^ and Q^2^ not larger than 0.3 to illustrate a robust model. VIP (Variable influence on projection) indicates the relevance of the group of X-variables that best explain Y. VIP predictive (VIPpred) was used if more than one component was identified in the OPLS/OPLS-DA model. We also used p(corr) that is the loading of each variable scaled as a correlation coefficient and thus standardizing the range from – 1 to + 1. To validate the model, we used cross validated analysis of variance (CV-ANOVA). A VIP-value ≥ 1.0 (or VIPpred ≥ 1.0), absolute p(corr) ≥ 0.5 and CV-ANOVA ≤ 0.05 were considered as significant. The analysis and the presented parameter in this study is in accordance with the guidelines presented by Wheelock and Wheelock^7,23^ and previously published studies.

## Results

### Background data

There was a statistically significant group differences in age (p = 0.003), systolic blood pressure (p = 0.05) and diastolic blood pressure (p = 0.002) (Table 2). The patient group consisted of 37.5% females and the healthy control group of 58.3% females (Table 2); the differences in proportions between groups was not statistically significant.

**Table 2:** Clinical variables for patients with neuropathic pain (NP) and healthy controls.

Variables	NP (n = 16) median (min–max)	Controls (n = 12) median (min–max)	p-value
Age	57.0 (39.0–75.0)	45.5 (21.0–55.0)	0.003
BMI (kg/m^2^)	27.10 (20.2–32.4)	24.2 (21.10–31.40)	0.35
Sex (% female)	37.5%	58.3%	0.283
Pain intensity (VAS, mm)	72 (40–94)	0 (0–0)	NA
Pain duration (months)	66 (12–300)	–	NA
Systolic blood pressure (mm Hg)	132.5 (117.0–182.0)	120 (105–145)	0.05
Diastolic blood pressure (mm Hg)	83.0 (71.0–121.0)	70.0 (65.0–80.0)	0.002

*VAS* Visual Analogue Scale, *NA* not applicable.

### Multivariate analysis of the metabolome in plasma from patients and controls

**Figure 1:**
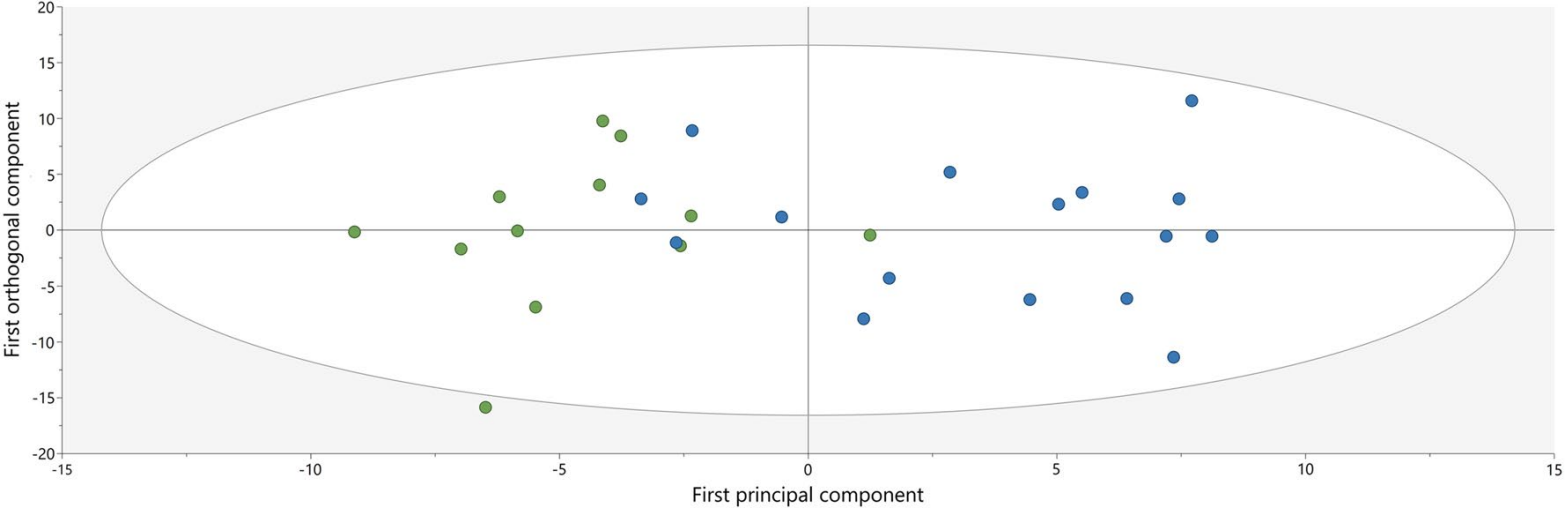
A score scatter plot depicting the subjects in each group (control group: n = 12, green dots; NP group: n = 16, blue dots) according to the Orthogonal Partial least square discriminant analysis (OPLS-DA) model based on metabolite concentrations in plasma. The horizontal axis shows the group separation while the vertical axis shows the within group separation.

An unsupervised PCA was conducted of the identified metabolites and showed no significant outliers. In Fig. 1, a significant separation of the patient and control group can be seen in a score scatter plot that is based on the OPLS-DA model (two components, R^2^ = 0.62, Q^2^ = 0.40, p = 0.016 by CV-ANOVA). The OPLS-DA analysis revealed 50 out of totally 327 features to be significantly associated with group membership at the VIPpred ≥ 1.0 and absolute p(corr) ≥ 0.5, which is illustrated in the loading scatter plot (Appendix 1) and listed in Table 3. To facilitate the interpretation of the obtained regression we also present univariate data; the Mann–Whitney *U* p-values illustrate which metabolites differed significantly in concentration between the two groups, while the VIP and p (corr) values describe if a metabolite showed a group correlation and, in that case, how much it contributed to the group separation (Table 3). Of these 50 plasma metabolites, 22 were unknown. Majority of the identified metabolites—whose levels differed significantly between the groups—were amino acids. The most important metabolite for the group discrimination was the amino acid histidine (VIP = 1.52, p(corr) = − 0.74, p-value = 0.004) that was decreased in NP. In addition, levels of serine, lysine and asparagine were also significantly decreased in NP compared to controls while levels of glutamine, phenylalanine, valine, and proline were increased in NP compared to controls. Lipids/free fatty acids, myo-inositol and choline and N-acetyl aspartate were also significantly elevated in NP compared to controls. Moreover, 4 of the metabolites listed in Table 3 were only detected in NP.

**Table 3:** Metabolites in plasma significantly differentiating between chronic neuropathic pain (NP) patients and healthy controls according to OPLS-DA.

Metabolites	VIPpred	p (corr)	Controls intensity × 1000 median (min–max)	NP intensity × 1000 median (min–max)	*p* value	NP vs con
his_26	1.52	− 0.74	140 (110–154)	118 (89–153)	0.004*	↓
NAAs + pro_313	1.47	0.72	502 (420–828)	893 (422–1772)	0.009*	↑
Unkn_358	1.45	0.71	1362 (848–2597)	2880 (632–10,151)	0.014*	↑
NAAs + pro_312	1.43	0.70	548 (367–801)	860 (391–2228)	0.016*	↑
his_9	1.42	− 0.70	131 (101–153)	115 (91–156)	0.011*	↓
lipid/ffa_36	1.40	0.69	330 (234–687)	710 (156–2162)	0.023*	↑
Unkn_355	1.39	0.68	137 (118–166)	167 (123–244)	0.008*	↑
Unkn_347	1.35	0.66	83 (57–174)	148 (47–548)	0.041*	↑
Unkn_24	1.33	0.65	0 (0)	18 (0–111)	0.001*	↑
Unkn_11	1.33	− 0.65	17 (13–19)	14 (12–16)	0.011*	↓
Unkn_25	1.32	0.65	0 (0)	20 (0–120)	0.001*	↑
NAAs + pro_309	1.32	0.64	385 (307–552)	533 (335–949)	0.026*	↑
Unkn_43	1.31	0.64	0 (0)	5 (0–36)	0.005*	↑
ser_86	1.31	− 0.64	179 (125–269)	153 (126–195)	0.041*	↓
Unkn_354	1.30	0.64	164 (142–208)	194 (143–285)	0.029*	↑
Unkn_44	1.28	0.63	0 (0)	6 (0–37)	0.005*	↑
NAAs + pro_315	1.28	0.63	330 (256–474)	416 (269–555)	0.037*	↑
Unkn_82	1.28	− 0.63	253 (207–305)	212 (155–267)	0.012*	↓
NAAs + pro_308	1.27	0.62	317 (239–411)	399 (289–648)	0.009*	↑
NAAs_310	1.27	0.62	1512 (1248–1835)	1697 (1277–2422)	0.02*	↑
Unkn_175	1.22	0.60	93 (83–116)	110 (70–165)	0.002*	↑
Unkn_215	1.21	0.59	121 (111–164)	143 (116–165)	0.002*	↑
Unkn_81	1.20	− 0.59	279 (210–332)	260 (166–289)	0.114	↓
Unkn_232	1.19	0.58	125 (99–147)	139 (103–223)	0.086	↑
Unkn_353	1.18	0.58	182 (162–234)	206 (164–290)	0.033*	↑
gln_290	1.18	0.58	147 (125–167)	164 (138–410)	0.006*	↑
Unkn_230	1.17	0.57	75 (53–114)	107 (41–251)	0.210	↑
NAAs + pro_305	1.17	0.57	175 (143–231)	203 (166–243)	0.007*	↑
NAAs + pro_316	1.16	0.57	222 (172–315)	274 (180–356)	0.043*	↑
gln_302	1.14	0.56	190 (173–213)	205 (187–230)	0.003*	↑
Unkn_373	1.13	− 0.55	38 (33–66)	35 (29–46)	0.095	↓
NAAs + pro_307	1.12	0.55	411 (318–494)	448 (362–574)	0.016*	↑
Unkn_352	1.12	0.55	132 (115–160)	143 (120–184)	0.033*	↑
myo-inositolcholine_76	1.11	0.55	82 (58–106)	95 (78–132)	0.005*	↑
pro + glu_271	1.11	0.54	74 (58–138)	102 (75–130)	< .001*	↑
Unkn_216	1.11	0.54	103 (94–130)	114 (102–136)	0.004*	↑
3-hydroxyisobutyrate or isobutyrate_372	1.11	− 0.54	97 (69–299)	74 (36–182)	0.057	↓
val_287	1.10	0.54	103 (78–147)	114 (87–395)	0.057	↑
phe_19	1.10	0.54	26 (21–32)	32 (25–107)	0.004*	↑
Unkn_80	1.10	− 0.54	206 (157–293)	179 (154–253)	0.086	↓
Unkn_218	1.10	− 0.54	115 (93–138)	104 (94–112)	0.114	↓
ser_84	1.10	− 0.54	294 (195–403)	258 (198–339)	0.07	↓
NAAs + pro_317	1.09	0.54	163 (126–234)	197 (128–265)	0.041*	↑
gln + unkn_304	1.08	0.53	159 (131–177)	171 (149–201)	0.029*	↑
ethanol_362	1.06	0.52	99 (92–117)	118 (97–183)	< .001*	↑
Unkn_56	1.06	0.52	88 (67–108)	93 (82–157)	0.104	↑
3-hydroxyisobutyrate or isobutyrate_370	1.05	− 0.51	89 (64–291)	72 (41–175)	0.078	↓
ser + mannose_87	1.04	− 0.51	191 (141–267)	169 (139–248)	0.078	↓
val_286	1.03	0.50	99 (76–127)	111 (88–230)	0.037*	↓
lys_213	1.03	− 0.50	182 (138–277)	175 (132–227)	0.286	↓

The metabolites are listed in descending order for VIPpred. A negative p (corr) indicates lower levels in patients while a positive p(corr) indicates the opposite. The p-value is according to Mann–Whitney test and *Refers to statistically significant differences between controls and NP patients. Up or down regulation is reported in the column furthest to the right. *His* histidine, *NAAs* N-acetyl aspartate, *Pro* proline, *Ser* serine, *Ffa* free fatty acid, *Unkn* unknown, *Lys* lysine, *Gln* glutamate, *val* valine, *phe* phenylalanine.

### Correlation analysis between metabolites and background data

In NP, multivariate correlation analyses (i.e., OPLS) were performed with age, BMI, systolic and diastolic blood pressure, VAS, and pain duration as dependent variables (Y). The results showed no effect of age and BMI on metabolite concentrations in plasma. The OPLS model with systolic blood pressure as dependent variable reached statistical significance (R^2^ = 0.74, Q^2^ = 0.37, one component (the predictive one), p = 0.048), see figure in Appendix 2. No significant multivariate correlations existed between the metabolites and the two pain variables (VAS and pain duration).

In the control group no significant multivariate correlation pattern existed between metabolites and age and BMI.

Multivariate correlation analyses were performed including all subjects (patients and healthy controls) using age, BMI, VAS, systolic and diastolic blood pressure as dependent Y-variables. The only significant model that was obtained was when VAS was chosen as dependent Y-variable (R^2^ = 0.66, Q^2^ = 0.39, two components, p = 0.02) (Appendix 3).

## Discussion

The major finding in this study were that several metabolites differ significantly in concentration in plasma between the NP patients and healthy controls. A number of 50 features were found to be significantly correlated to group membership in plasma.

According to univariate statistics, the parameters age, and blood pressures (systolic and diastolic) significantly differed between the two groups. It is reasonable to believe that age, BMI, and blood pressure are interrelated to a certain extent. When looking at all individuals together (patients and controls), no multivariate correlation by OPLS could be found between these parameters and metabolites. By contrast, other studies have showed a correlation between several of the identified metabolites in this study and BMI, and correlations to age. Higher levels of asparagine, histidine and serine were associated with higher BMI, while higher levels of tyrosine, glutamate and choline are correlated negatively with BMI^24^. In this study we detected lower levels of histidine in NP patients showing that histidine might be play a role in the pain mechanism in NP patients independently of BMI. When it comes to age, lower levels of histidine and serine have been related to higher ages^25^. No significant correlation could be identified between the metabolome and pain intensity measured as VAS among the patients with NP in this study.

The metabolites of interest in plasma, which could discriminate patients from healthy controls were histidine, N-acetylated amino acids such as N-acetyl aspartic acid (NAA), proline, serine, lipid/free fatty acids, myo-inositol + choline, lysine, and asparagine. Histidine is a precursor for synthesis of histamine and serves as an antioxidant and anti-inflammatory metabolite^26^. Its lower levels in patients with neuropathic pain could indicate a decreased anti-inflammatory activity. NAA is one of the most concentrated metabolites in the brain. It has multiple important functions (many of them still unknown), but the most critical functions are regulation of the fluid balance in the brain, providing acetate to oligodendrocytes so that they can synthesize myelin and being a precursor for the neurotransmitter N-acetylaspartylglutamate (NAAG). NAAG is an agonist at mGluR3 receptors and antagonist at NMDA-receptors. High levels of NAA can cause abnormal neural signaling and be neurotoxic^26^. The patients in this study had remarkably higher plasma NAA concentrations than the controls; it is not unlikely to believe that this can affect the nociceptive neural signaling. But on the other hand, several previous MRS studies on brain regions such as the thalamus and ACC have showed decreased levels of NAA in chronic neuropathic pain patients which was thought to be associated with neuronal injury and dysfunction of inhibitory neurons^18,19^. Certainly, differences in the methodology used and inclusion of other chronic neuropathic pain patient groups could contribute to these discrepancies.

Serine is derived from glycine and counts as a non-essential amino acid. It is found in high concentrations in cell membranes and plays an essential role in cell proliferation. Also, serine is important for different functions of the central nervous system^26^, which at lower levels that is seen in this study among patients potentially could lead to CNS malfunction. However, the concentration differences between patients and controls were not prominent even though significant.

Myo-inositol is a so-called sugar alcohol that is synthesized from glucose, mainly in the kidneys. This metabolite acts as a precursor to various second messengers in cells^26^. It was seen in higher concentrations, in combination with choline, in the NP group (Table 3). Choline is primarily found in phospholipids, more specifically it is a constituent of phosphatidylcholine or lecithin. It is a precursor of acetylcholine and has an important role in lipid metabolism^26^. An MRS study on selected brain regions on different chronic neuropathic pain syndromes has showed elevated levels of the glial metabolites myo-inositol and to a lesser degree choline compounds in brain regions such as thalamus and ACC, which can indicate an activation of microglia and astrocytes and neuroinflammation in these regions^18^.

Lysine is an essential amino acid that, in comparison to other amino acids, is highly concentrated in muscles. Stress increases the consumption of this amino acid. Low levels of this amino acids have been found in patients with depression, kidney disease, hypothyroidism, and Parkinson’s disease^26^ and now also in the NP patients of this study. The lower levels in NP could be a consequence of pain-induced stress leading to more lysine consumption. Asparagine is a non-essential amino acid and oxaloacetate is its precursor, it is involved in cell functioning in the brain and nervous system^26^. Decreased levels could hypothetically affect the nervous system.

The tetrahydrobiopterin (BH4) and Kynurenine (KYN) pathways are two metabolic pathways that are thought to be activated by neuroinflammation and lead to the production of different bioactive metabolites, such as BH4 and quinolinic acid (QUIN; a KYN pathway metabolite), contributing to chronic pain states by inducing neuronal sensitization^8^. The hypothesis that activation of the metabolic pathways KYN and BH4 during inflammation occurs has been demonstrated and supported in various human studies. Significant serum increases of the biomarkers neopterin (metabolite of the BH4-pathway) and KYN/TRP ratio (indirect marker of the enzymatic activity of IDO1 that is a major enzyme in the KYN pathway converting tryptophan [TRP] to KYN) owing to the lower levels of TRP and higher KYN levels has been detected in patients with diabetic neuropathic pain. This study also showed elevated levels of cytokines IL-8 and GM-CSF in the patient group^8^.

This study design was based on NMR metabolomics. Not many similar NMR metabolomics studies on neuropathic pain have been done prior to this study. Currently, the previous studies have either included patients with another type of pain and/or examined other biofluids. Hence, in an NMR metabolomics study aimed to identify differences in the global urine metabolic profile between patients with neuropathic pain, nociceptive pain and pain-free controls, elevated levels of phosphocholine, alanine and taurine among the neuropathic pain patients were found^16^. Elevated levels of choline and phosphocholine can be seen in conditions with demyelination, gliosis, and cell membrane turnover. Alanine and taurine can be regarded as markers of apoptosis and are released during neural cell damage or stress^16^. In this study only elevated levels of choline in combination with myo-inositol was detected in the plasma of patients. In another NMR urine metabolomics study of female fibromyalgia (FMS) patients (which also is a chronic pain syndrome but with a nociplastic characterization) an altered metabolic profile was detected within their urine when compared to 3 control groups. Elevated levels of the endogenous metabolites taurine, succinic acid and creatine were most significant for the separation of FMS patients and controls, and they also showed a correlation to pain and fatigue symptoms. Additionally, that study observed significant increases in gut microbiome related metabolites hippuric acid, 2-hydroxyisobutyric acid and lactic acid which possibly can indicate effects on the gut-brain axis among FMS patients^27^. The metabolic profile in serum of female patients with rheumatoid arthritis have also been studied with NMR, showing 12 metabolites that differentiated the RA patients from healthy controls. Valine, isoleucine, lactate, alanine, creatinine, sn-glycero-3-phosphocholine + acetylphosphocholine (GPC + APC, which are derivates of choline) and histidine were decreased in RA patients. Ketone bodies such as 3-hydroxyisobutyrate, acetate, NAC (N-acetylated glycoprotein), acetoacetate and acetone were elevated^17^.

This study, like most other metabolomics studies, was limited by the few numbers of subjects that were available for investigation. Follow-up metabolomics research in larger chronic neuropathic pain cohorts is required to further investigate the metabolome and validate findings presented in this study. Also, it is important to further identify all the unknown metabolites. In future studies, it will be important to age-match the controls and the patients to eradicate a possible confounding effect of age. The same applies to gender matching. Also, a larger control group should be recruited in future studies. A slightly significant correlation was found for systolic blood pressure and the metabolites, which is needed to be investigated in larger cohorts of patient to conclude if the high blood pressure is confounding factor that might contribute to the identified differences in metabolome profile in this study. Future study with large sample size where patients with and without comorbidities are included, is highly warranted.

## Conclusions

In this study we investigated the metabolic profile of patients with chronic neuropathic pain in plasma, where 20 metabolites showed a correlation to group membership. The metabolite levels in plasma showed no correlation to pain intensity among the neuropathic pain patients. These preliminary results possibly indicate that there are differences in the metabolic profile between patients with chronic neuropathic pain and healthy individuals. Several of the metabolites that significantly differed between the patients and controls were involved in inflammatory processes, while others were important for CNS functioning and neural signaling. These findings are preliminary first steps towards increasing our knowledge about the pathophysiological mechanisms underlying chronic neuropathic pain. We consider exploratory studies such as this one as being part of a paradigm change towards more of precision medicine in chronic pain care.

## Supplementary Information

Supplementary Information 1.

Supplementary Information 2.

Supplementary Information 3.

## Data Availability

The datasets generated and/or analyzed in this study are not publicly available as the Ethical Review Board has not approved the public availability of these data.

## References

[1] van Hecke O (2014). Neuropathic pain in the general population: A systematic review of epidemiological studies. Pain.

[2] Colloca L (2017). Neuropathic pain. Nat. Rev. Dis. Primers.

[3] Attal N (2011). The specific disease burden of neuropathic pain: Results of a French nationwide survey. Pain.

[4] Bouhassira D, Attal N (2019). The multiple challenges of neuropathic pain. Neurosci. Lett..

[5] Finnerup NB (2015). Pharmacotherapy for neuropathic pain in adults: A systematic review and meta-analysis. Lancet Neurol..

[6] Sommer C, Leinders M, Üçeyler N (2018). Inflammation in the pathophysiology of neuropathic pain. Pain.

[7] Jönsson M (2021). The inflammatory profile of cerebrospinal fluid, plasma, and saliva from patients with severe neuropathic pain and healthy controls-a pilot study. BMC Neurosci..

[8] Staats Pires A (2020). Kynurenine and tetrahydrobiopterin pathways crosstalk in pain hypersensitivity. Front. Neurosci..

[9] Calvo M, Dawes JM, Bennett DL (2012). The role of the immune system in the generation of neuropathic pain. Lancet Neurol.

[10] Bates D (2019). A comprehensive algorithm for management of neuropathic pain. Pain Med..

[11] Kuffler DP (2020). Injury-induced effectors of neuropathic pain. Mol. Neurobiol..

[12] Wu W, Ji X, Zhao Y (2019). Emerging roles of long non-coding RNAs in chronic neuropathic pain. Front. Neurosci..

[13] Meacham K (2017). Neuropathic pain: Central vs peripheral mechanisms. Curr. Pain Headache Rep..

[14] Bäckryd E (2015). Multivariate proteomic analysis of the cerebrospinal fluid of patients with peripheral neuropathic pain and healthy controls: A hypothesis-generating pilot study. J. Pain Res..

[15] Teckchandani S (2021). Metabolomics in chronic pain research. Eur. J. Pain.

[16] Finco G (2016). Can urine metabolomics be helpful in differentiating neuropathic and nociceptive pain? A proof-of-concept study. PLoS ONE.

[17] Zabek A (2016). Application of (1)H NMR-based serum metabolomic studies for monitoring female patients with rheumatoid arthritis. J. Pharm. Biomed. Anal..

[18] Chang L (2013). Magnetic resonance spectroscopy to assess neuroinflammation and neuropathic pain. J. Neuroimmune Pharmacol..

[19] Fukui S (2006). N-Acetylaspartate concentrations in the thalami of neuropathic pain patients and healthy comparison subjects measured with (1)H-MRS. Magn. Reson. Imaging.

[20] Bäckryd E, Sörensen J, Gerdle B (2015). Ziconotide trialing by intrathecal bolus injections: An open-label non-randomized clinical trial in postoperative/posttraumatic neuropathic pain patients refractory to conventional treatment. Neuromodulation.

[21] Jönsson M (2022). Differences in plasma lipoprotein profiles between patients with chronic peripheral neuropathic pain and healthy controls: An exploratory pilot study. Pain Rep..

[22] Wishart DS (2022). HMDB 5.0: The human metabolome database for 2022. Nucleic Acids Res..

[23] Wheelock ÅM, Wheelock CE (2013). Trials and tribulations of 'omics data analysis: assessing quality of SIMCA-based multivariate models using examples from pulmonary medicine. Mol. Biosyst..

[24] Moore SC (2014). Human metabolic correlates of body mass index. Metabolomics.

[25] Yu Z (2012). Human serum metabolic profiles are age dependent. Aging Cell.

[26] Wishart DS (2018). HMDB 4.0: The human metabolome database for 2018. Nucleic Acids Res..

[27] Malatji BG (2017). A diagnostic biomarker profile for fibromyalgia syndrome based on an NMR metabolomics study of selected patients and controls. BMC Neurol..

