# Supplementary figures and images for: An investigation of metabolome in blood in patients with chronic peripheral, posttraumatic/postsurgical neuropathic pain
Source: Sci Rep. 2022 Dec 15;12:21714. doi: 10.1038/s41598-022-26405-6 (PMC9755304; doi:10.1038/s41598-022-26405-6)

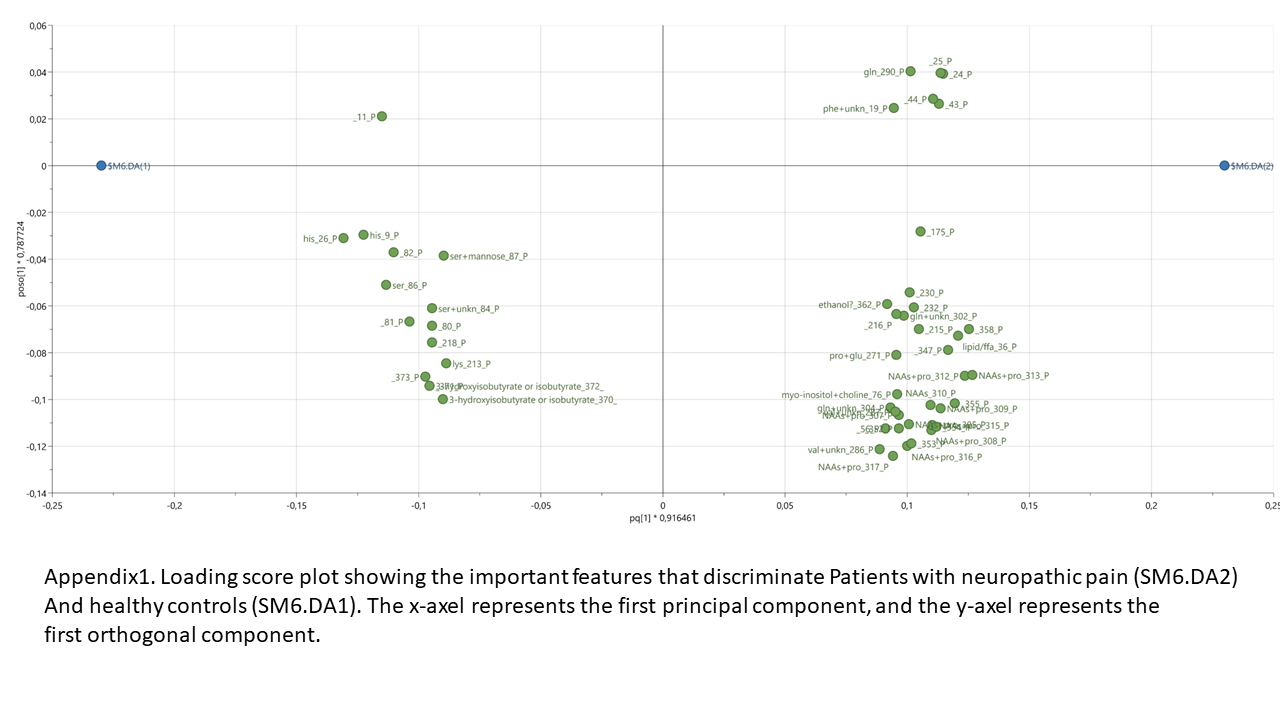

Supplement: Supplementary file 1 — Supplementary Information 1. [file 41598_2022_26405_MOESM1_ESM.tif]

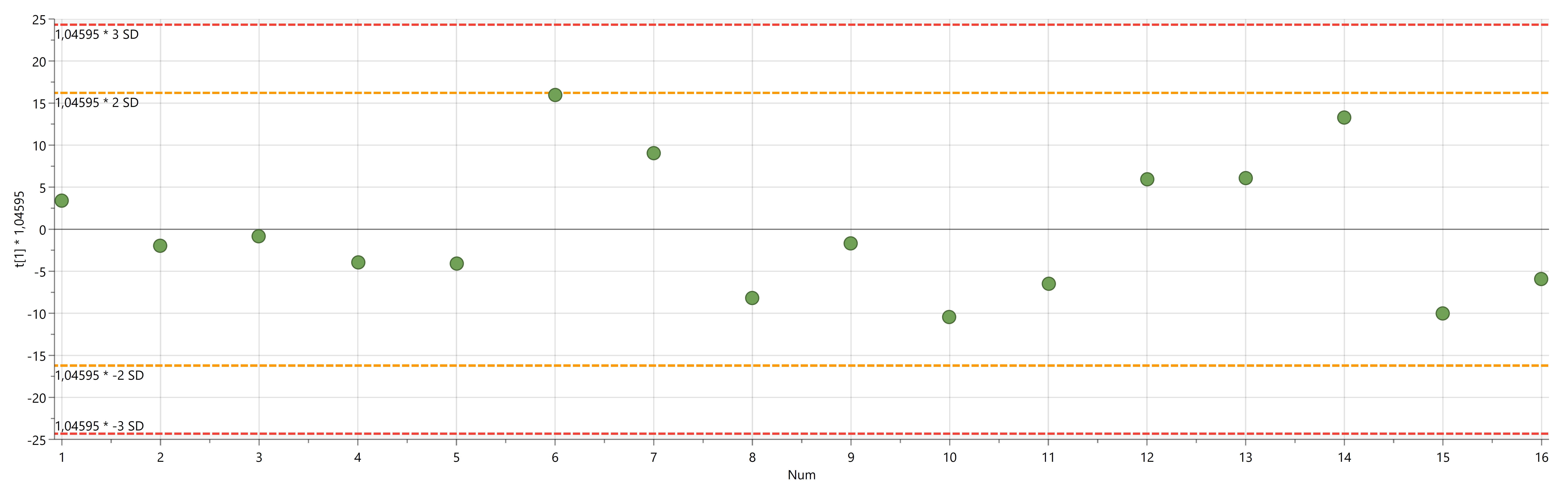

Supplement: Supplementary file 2 — Supplementary Information 2. [file 41598_2022_26405_MOESM2_ESM.jpg]

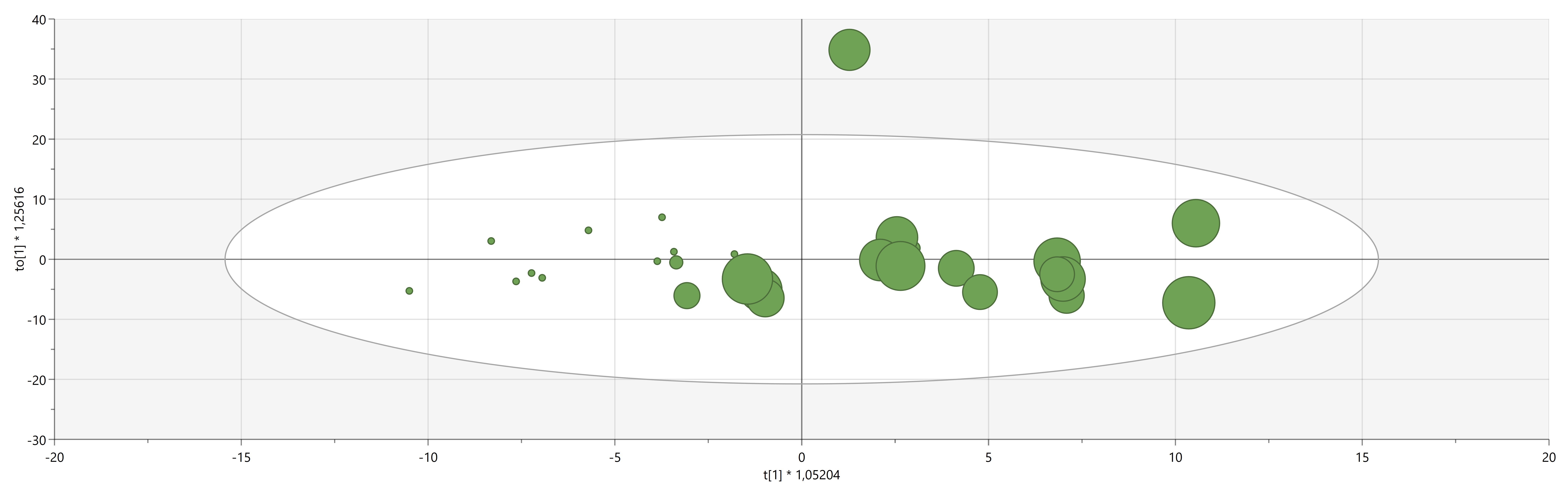

Supplement: Supplementary file 3 — Supplementary Information 3. [file 41598_2022_26405_MOESM3_ESM.jpg]
